# Some Affections of the Gums

**Published:** 1888-09

**Authors:** Frank Lankester

**Affiliations:** England.


AMERICAN JOURNAL

—OF—

DENTAL SCIENCE.

VOL. XXII. THIRD SERIES—SEPTEMBER, 1888. No. 5.

ARTICLE I.

### SOME AFFECTIONS OF THE GUMS.

BY FRANK LANKESTER, L. R. C. P., M. R. C. S., L. D. S. ENGLAND.

I shall endeavor, so far as time will permit, to bring before your notice some of the chief and more important affections of the gums, including those that usually come under the care of the general practitioner, and I shall hope to show how very important it is that we, as dentists, should be thoroughly well acquainted with them.

We of all men should know what is the normal healthy appearance of the gums, and should therefore also at once notice any abnormal condition that they may present. In examining a patient's mouth for caries, or any other diseased condition, we do well to cultivate a habit of thoroughness; we should not be satisfied with merely looking at the teeth, but should be careful also to inspect the gums, for we may often learn much about the condition of a tooth or root by simply observing the state of the surrounding parts. Then, if anything abnormal be present, we should endeavor to find out the cause of it and, if possible, a remedy for the same.

I thought the following was, perhaps, the best *classification* of the different affections, and I must thank my friend Mr. H. Arthur Sansom for some help in this matter, to whose kindness we are also indebted for the loan of the various diagrams I shall send round.

We may broadly divide the affections into two classes; the LOCAL and the CONSTITUTIONAL.

The *Local*, consisting of those which originate in the gums themselves, include the following:—

1. Simple inflammations from injury, &c.
2. Parulis, gumboil or alveolar abscess.
3. Simple epulis, or fibroma.
4. Polypus of gum, or simple hypertrophy.
5. Papillary and warty growths.
6. Vascular tumors, or nævoid growths
7. General hypertrophy.
8. Pyorrhœa alveolaris (so-called Riggs' disease); and
9. Primary epithelioma, or cancer (carcinoma).

The second class, the *Constitutional*, consist of those which occur in the course of a general systemic disorder, and include—

1. Syphilis.
2. Scrofula.
3. Scurvy.
4. Purpura hæmorrhagica.
5. Addison's disease.
6. Stomatitis, of which there are three forms: simple; ulcerative; gangrenous (noma).
7. Aphthæ, or thrush.
8. Herpes; and
9. Lastly, the metallic poisons; lead, silver, mercury and copper.

We should certainly be able to form a correct diagnosis of any case we may meet with, though to do this often requires considerable practical experience as well as mere book knowledge. Many of these affections we, as dentists, are often called upon to treat, and there are others which, having diagnosed, we should hand over to the care of the general practitioner. These latter, which are generally the more serious cases, we may from time to time, perhaps quite accidentally, meet with, and we should be just as well acquainted with them as we are with the simpler affections. This, too, should be the more insisted on, as we are more likely to see them in their earlier stages, and by a proper and immediate appreciation of the diseased condition we may be able to warn our patient, and thus induce him to obtain prophylactic treatment, and thereby perhaps save him from a long and serious, nay even fatal, illness.

Before we now proceed to discuss some of these affections, it would perhaps be well for me to first very briefly remind you of the chief points in the *structure* of the gums. They are mainly composed of a dense fibrous connective tissue, cohering very closely with the alveolar and dental periosteum, whilst on their surface they are covered with a very vascular mucous membrane: this is mostly smooth, but, in the neighborhood of the necks of the teeth, it is beset with very fine papillæ.

With these opening remarks, I will now try to place before you as briefly as possible a few observations on most of the various affections which have been enumerated, and we will first consider the *local* ones, commencing with—

*Parulis or Gumboil.*—The external manifestation on the gum of alveolar abscess. This affection consists of an inflammation usually running on to suppuration, occurring about the apex of the root of a tooth. The apex itself becomes enclosed in the abscess sac, the latter being adherent to the root at a little distance from the apex. The dental periosteum is included in the sac walls. The tooth pulp is nearly always dead, and it is the decomposition of the dead pulp that generally gives rise to the abscess. The dental periosteum first becomes inflamed at the apex, suppuration soon takes place, and the pus that forms strips off the membrane from the root, and an alveolar abscess is the result. When there is no exit for the pus through the pulp canal, it finds an exit for itself by "pointing" externally, and generally on the gums, as the so-called gumboil. In rare cases simple periostitis from cold, &c., may go on to suppuration, and so be the cause of parulis. The pus soon finds its way through the alveolar plate, and appears beneath the gum immediately (as a rule) over the diseased root as a small, red, tense, very tender swelling. There is always a good deal of pain, it being generally worse at night when the recumbent posture is assumed. Pain is of a throbbing character. There is always a certain amount of ostitis and periostitis around the seat of inflammation, so that gentle percussion on the adjacent teeth will cause pain, though of not nearly so severe a degree as will be that caused by similarly percussing the tooth actually connected with the abscess. This difference in tenderness will help us to decide which is the offending tooth. If left alone, the pus will come to the surface and discharge itself, leaving behind it a sinus which frequently remains open for months.

At the orifice of the sinus, little sprouting granulations often appear, indicating the site of a former gumboil which has discharged itself in this way.

As regards treatment, I cannot do more than just mention that in the early stages before suppuration has taken place complete relief may often be afforded by painting the inflamed area two or three times with the liniment of iodine, but before doing this it is very important to first thoroughly dry the gums previous to applying the iodine. as otherwise the latter simply floats as it were on the moist mucous membrane, without affecting it very materially. A good saline purge should also be administered. This will often be found very useful in the case of a recently stopped tooth which appears to be "going wrong," but which you do not wish to interfere with if you can possibly avoid so doing. The tincture of iodine is much too weak a preparation to be of much good. We must not forget the possible results of alveolar abscess if neglected or improperly treated. External poulticing (the usual resort) will frequently cause it to point on the cheek externally. I had a case only two weeks ago in which there had been a fistulous opening in the cheek for months, and a probe passed right through into the mouth; I removed a root and the sinus healed up immediately. When suppuration, therefore, has set in, relief can and should at once be given by evacuating the pus, either by a small lancet or by the extraction of the offending root.

*Superficial Gumboil.*—Salter mentions that there is another form of parulis occasionally met with which is associated with loose teeth, absorbed sockets, and tumid gums. The pus is situated in the substance of the latter, but quite superficial to the alveolar processes.

The next affection we have to consider is *Epulis*. This term has been vaguely applied to various tumors in and upon the gums (a fact to be explained by the etymological meaning of the word, *epi, ouli*, upon the gums). But by the simple or true epulis we mean those dense fibrous tumors that arise upon the surface of the alveolus and involve the periosteum, and by their growth cause the overlying gum to become stretched over them, the gum being otherwise healthy. They differ in origin, history and structure from those pendulous masses so often found around decayed teeth and known as polypus. There is no connection between epulis and caries as is the case with polypus and caries. A true epulis, then, is a hard dense mass of fibrous tissue, starting from the alveolar margin, usually between two teeth and more generally on the labial than on the lingual aspect. It is usually seen upon that part of the gum projecting up between the necks of two teeth, one or more of the latter being usually displaced by its growth, which is slow and regular. It increases more in its basal area than in its vertical diameter, starting from the porous and vascular alveolus and being closely connected with the periosteum usually by a narrow pedicle. The gum over it is usually healthy, but it may be mottled and slightly lobulated. The tumor itself is tense and elastic to the touch, insensitive and of low vascularity. Its size varies from that of a pea to that of a walnut and it is about twice as often met with in the upper as it is in the lower jaw. It occurs usually in connection with some one particular tooth, the removal of which, together with the excision of the tumor, nearly always results in a complete and immediate cure. It is very rarely seen in an edentulous jaw, and then it will generally be found to be connected with some hidden stump. Salter relates a good case in illustration of this, in which a tumor repeatedly recurred after excision until the real cause was discovered. The epulis extended from the canine to the wisdom tooth, the four intermediate teeth having been removed. It was excised again and again, but always recurred until on one occasion a first molar stump was discovered and extracted, after which there was no further recurrence. The alveolar periosteum, the endosteum and the fibrous tissue of the gum are all continuous, and all share alike in the development of this growth. They are perfectly innocent and painless tumors, but they may take on a malignant character.

As to the treatment. In an ordinary case it will be well to first try the effects of simple removal by excision with a scalpel, together with a small portion of the spongy bone at the base of the growth. If it returns, as is frequently the case, repeat the operation together with the extraction of one or more of the implicated teeth. The alveolus will thereby become absorbed and will no longer form a nidus for the recurrence of the tumor. When arising deeply from the socket of a tooth, it may then be necessary to cut away a V-shaped portion of the alveolus, together with a very small piece of the subjacent bone. This can easily be done with a Hey's saw and a pair of bone nippers. Any hæmorrhage can easily be checked by the actual cautery. If the growth be unrestrained by treatment, it will continue to enlarge and interfere with articulation and mastication, and thereby causes much inconvenience. The surface but rarely ulcerates, but it may be injured by opposing teeth; and ulceration thus excited may give rise to a copious and fœtid discharge. Sometimes sarcomatous cells are mixed up with the fibrous tissue, and we may then expect it to recur after removal.

*Polypus* or *Simple Hypertrophy* of the gum comes next on our list. This is purely a local affection of the gums alone, quite apart from the periosteum. It is generally associated with uncleanly habits, the presence of tartar or some other form of irritation, and is usually most marked along the borders or margin of the gums, and especially the interdental portions. It consists in a simple hypertrophy of the gum tissues, and is brought about by some long-continued irritation. The gums may be so increased in size as to nearly cover the crowns of the teeth, and it is most commonly met with, and most pronounced, in the front part of the mouth. These two models I now send round are very good examples of this affection. They were lately presented to our society by Mr. Jones. The treatment as a rule is very simple, and consists in scarifying the gums and getting rid of the irritating cause, *i. e.*, by thoroughly cleansing the teeth, removing all tartar, and washing the mouth occasionally during the day with a strong solution of tannin or other harmless astringent, to which may be added a very small quantity of Condy's fluid. The gums will then, usually, soon recede to their normal size and condition. I saw a case, yesterday, which Mr. Humby treated for a long time before curing his patient. This was caused in first instance by wearing a regulation plate. The hypertrophy was so extensive and rapid that the wearing of the plate had to be discontinued.

A more truly polypoid form of this same affection of the gum may be very frequently seen in connection with almost any large cervical cavity. It occurs as a small pedunculated mass, resembling the gum tissue, and more or less fills up the cavity. It is due to the constant irritation caused by the rough edge of the cavity coming into contact with the gums. The irritation leads to a slight chronic inflammation and consequent overgrowth of the existing gum tissues (*i. e.*, hypertrophy), in which the true mucous membrane elements take part. The tumor is usually tender and at times painful. The treatment, if any be required, consists in removing the cause, either by filling the cavity or getting rid of the tooth or root with its projecting sharp edge. When nothing more than simple excision of the growth is performed, it almost always returns. This is well illustrated in a case which Salter relates, in which the hypertrophied mass was about half as large as a chestnut, and much resembled ordinary gum in appearance. At first sight it might very easily have been mistaken for an epulis. It arose from the portion of gum situated between the three separated roots of an upper first molar and was attached by a short pedicle. It was first excised, the stumps were then removed, and there was, of course, no recurrence.

*Papillary Growths* come next on our list. These may occur on the gums, but are exceedingly rare. They consist simply of hypertrophied papillæ, and are really unimportant. Caustics sometimes arrest or cure the growths, but a free excision is usually the best treatment, though even after this they sometimes tend to recur.

*Warty Growths* are also very rarely met with on the gums, but they do occur, and much resemble the ordinary cauliflower excresences so frequently seen on the hands, &c. They are quite innocent, but there is often very considerable difficulty experienced in getting rid of them, just as is the case when they occur on the hands. At the same time, there is the liability for them to take on a malignant character. They are of a pale whitish color, and contrast very strongly with the surrounding red gum. The microscope-speculum I now pass round shows their structure. It is really a papilloma of the neck.

*Vascular Growths.*—Another very uncommon affection is a nævoid or vascular tumor of the gum. Such usually occur in adult or middle life, and are most commonly situated in the upper jaw, between the incisors and canines. They are generally of small size, about as large as a pea, and are often more or less pedunculated. They tend to increase in size; they are compressible, and can thus be reduced to the level and color of the gums. They are of a bright red color, and bleed readily on pressure, &c. Hæmorrhage is indeed their most important symptom; it usually occurs at night, when in the recumbent posture, and consists in a general oozing from the whole surface of the growth. In rare cases this may be very severe, and on this account they may require removal, though they are perfectly innocent, and do not return when carefully eradicated. This is best done by freely excising with the knife, including, at the same time, a small portion of the subjacent vascular and spongy bone. Cold and pressure will usually check any hæmorrhage. Any subsequent sprouting granulations should be touched with nitrate of silver. Before resorting to excision, you might first try the effects of ligaturing the growth, or of destroying it by caustics.

The condition known as *General* or *Congenital Hypertrophy* of the gums and alveolus is exceedingly rare. It occurs chiefly in children, and with it is frequently associated some other tegmentary hypertrophy, such as a thick skin, coarse hair and nails, &c. The whole or the greater part of the gums and alveolar processes are involved in a general hypertrophy. The greatly enlarged alveolar processes are covered by the much thickened gums; the latter being pale, inelastic, firm and insensitive. The growth may increase to such an extent as to prevent the perfect closure of the lips. The teeth are often quite hidden; especially is this so in the front of the mouth, where the condition is always more marked. Both jaws are usually affected. The thickening of the gums is due to an immense increase of the fibrous tissue, together with an enormous enlargement of the papillæ of the mucous membrane. The teeth, too, are of an immense size comparatively. The progress of the disease is slow and unaccompanied by pain, nor is there any tendency to ulceration. The treatment consists in paring off the lobular masses of the gum, and then in excising the hypertrophied alveolus with a pair of bone nippers. The whole of the growth need not be removed in one operation. There is but slight tendency for it to recur. This treatment involves, of course, the loss of the teeth, but they are worse than useless so long as the hypertrophy exists. I am indebted to Mr. Weiss for these models of this affection.

We will now pass on to consider an obscure, though somewhat important affection, known as Riggs' disease, or a better and more correct name is *Pyorrhœa Alveolaris*. It is not so very uncommon, and consists in a rapid and premature loss of the alveolar processes, together with a certain amount of inflammatory disturbance. One of the first indications of the advent of this disease is seen in a thickening and rounding of the margins of the gums on both sides of the mouth, and they cease to be closely adherent to the necks of the teeth. Between the gums and cementum for a short distance there is generally a little pus to be found. The breath is fœtid or nauseating, and there is a considerable amount of offensive discharge.

Neuralgic pain is often present, as also a chronic inflammation of the gums. It sometimes follows scurvy and mercurialism. The causes and pathology of the disease are very obscure, but there are a good many reasons that point to its being a part of a constitutional disease. Treatment is very far from successful in curing this affection, and it rarely does more than temporarily retard the progress of the disease. It consists in first of all removing very carefully and thoroughly all tartar from the necks of the teeth and from within the margin of the gums, and as the seat of the disease is probably at the edges of the alveolar processes, it is sometimes very beneficial to scrape these processes; it, however, gives considerable pain. Some strongly advocate the use of caustics, &c., to be applied to the edges of the gums, and to be rubbed well up towards the necks of the teeth within the gum margins. The following are those most in vogue—Powdered sulphate of copper, iodoform, aromatic sulphuric acid, chloride and iodide of zinc. As the disease progresses, the alveolus becomes more and more absorbed, the teeth get loose and finally fall out.

We now pass on to about the last, though by no means the least important, of the local affections of the gums; in fact, so far as the patient is concerned, it is by far the most important of them all, and such is the point of view in which it should also be regarded by the dental practitioner, though it may not come under his notice nearly so often as some of the other affections. He may perhaps never meet with a case in his own practice, and yet I hold that he should be thoroughly well able to diagnose it should a case come under his notice or care. I refer, as you know, to *epithelioma* or *cancer* and sometimes *wrongly* called *malignant epulis.* The latter term should only be applied to those myeloid sarcomatous tumours of the jaw that Mr. Heath has described. I have not time to say anything more about this latter affection, and it hardly comes within our category to-night, but there is a very typical specimen of one under the microscope in which you can well see the large myeloid or giant cells scattered about amongst the smaller spindle-shaped sarcomatous cells. Towards the surface are a few fragments of the expanded bone which appear of a yellowish colour; you will observe a great difference between this specimen and the fibroma, which you have already seen.

After this slight digression we will return to the consideration of *epithelioma* originating in the gums. It is generally due to some long-continued irritation such as that excited by the wearing of an ill-fitting artificial denture, or the presence of unhealthy roots, to which may often be added a strong hereditary predisposition to cancer. I don't think we should lose sight of this fact when dealing with artificial work. Any ulceration of the gums or mucous membrane of the mouth occuring in an elderly patient, and which does not readily yield to treatment, but rather tends to grow worse, should be looked upon with very grave suspicion as to its nature. These cases are generally first seen by a dentist, and often at a comparatively early stage of the disease, at a time when it may be a matter of life and death to the patient that the disease be recognized at once and properly treated. I fear that if such a case at an early stage of the disease were presented to the average dentist of to-day it would in but too many instances merely receive palliative and expectant treatment; meanwhile the disease would go on steadily but surely progressing, valuable time would be lost, and when at last the dentist hands it over to the care of the surgeon, all hope of a radical cure may be gone for ever, or at least be much more doubtful. Hence the great importance of the dental practitioner being thoroughly well acquainted with the disease, its diagnosis, and serious prognosis. It generally occurs in persons over forty years of age, and usually in the form of a warty or papillated growth, which ere long tends to ulcerate and break down in the centre of its free surface, whilst it continues to spread circumferentially, by invading the surrounding tissues. There is great induration of these tissues all round the growth. The central ulcerating portion soon becomess deeply excavated, the edges become raised and everted, ragged and irregular, and considerably undermined. The surface is sometimes very vascular, and bleeds readily on the gentlest touch. It has adirty unhealthy appearance, is of a greyish colour, and there oozes from it a little thin sanious discharge. The growth soon involves the adjacent tissues, such as the cheek, tongue, &c., the teeth in the affected area become loose and fall out, and it is not very long before the submaxillary lymphatic glands in the neck become secondarily affected. There is generally more or less pain, and sometimes it is very severe. The lower jaw is more frequently affected than the upper. Mr. Christopher Heath says that nothing but very free and early removal offers the patient a chance of permanent relief, and that it is much better to remove a piece of the whole thickness of the jaw well beyond the seat of the disease rather than merely the alveolar margins, since you can never tell how deeply the bone may be involved. This leads necessarily to permanent disfigurement, but by doing less you are running a very serious risk of its recurrence. It is, of course, a case for a surgeon to treat, but it is occasionally the dentist's duty to diagnose and hand over such a case to the surgeon.—*London Dental Record.*

